# Virtual meetings and social isolation in COVID-19 times: transposable barriers

**DOI:** 10.1590/2237-6089-2020-0065

**Published:** 2020-10-08

**Authors:** Vicente Sperb Antonello, Alana Castro Panzenhagen, Vicent Balanzá-Martínez, Flávio Milman Shansis

**Affiliations:** 1 Serviço de Controle de Infecção Hospitalar Hospital Fêmina Porto AlegreRS Brazil Serviço de Controle de Infecção Hospitalar, Hospital Fêmina, Porto Alegre, RS, Brazil.; 2 Programa de Pós-Graduação em Ciências Biológicas Universidade Federal do Rio Grande do Sul Porto AlegreRS Brazil Programa de Pós-Graduação em Ciências Biológicas: Bioquímica, Universidade Federal do Rio Grande do Sul (UFRGS), Porto Alegre, RS, Brazil.; 3 Centro de Pesquisa Translacional em Transtornos do Humor e Suicídio Universidade do Vale do Taquari LajeadoRS Brazil Centro de Pesquisa Translacional em Transtornos do Humor e Suicídio (CEPETTHS), Universidade do Vale do Taquari (Univates), Lajeado, RS, Brazil.; 4 Departamento de Medicina Universidad de València Centro de Investigación Biomédica en Red de Salud Mental València Spain Unidad Docente de Psiquiatría y Psicología Médica, Departamento de Medicina, Universidad de València, Centro de Investigación Biomédica en Red de Salud Mental (CIBERSAM), València, Spain.; 5 CIBERSAM Instituto de Salud Carlos III Madrid Spain CIBERSAM, Instituto de Salud Carlos III (ISCIII), Madrid, Spain.; 6 Programa de Pós-Graduação em Ciências Médicas Univates LajeadoRS Brazil Programa de Pós-Graduação em Ciências Médicas, Univates, Lajeado, RS, Brazil.; 7 Programa de Pós-Graduação em Ginecologia e Obstetrícia UFRGS Porto AlegreRS Brazil Programa de Pós-Graduação em Ginecologia e Obstetrícia, UFRGS, Porto Alegre, RS, Brazil.

Social isolation has been shown to severely impact the health of isolated individuals and has been associated with stress, anxiety and mood disorders. Technological correspondence has been pointed out as one of the greatest villains of generation Z times, in which loss of in-person communication between individuals has become a fairly common phenomenon. Specialists attribute many communication and affective impairments to the increasing use of social media and virtual interactions.^1 , 2^

Currently, however, in times of coronavirus disease 2019 (COVID-19), we are experiencing an unprecedented crisis, affecting a total of 213 countries and territories around the world.^3^ Additionally, the public is stunned by news that generate fear and anxiety, and may result in psychological dysregulations, e.g., stress-related disorders.^4^ The recommendation of social distancing measures aims at diminishing the disease spread and preventing the chain of viral transmission. Notwithstanding, there is an incentive to social connection through virtual media within families and communities.^3^ So, can virtual groups go from villains to problem-solvers?

Virtual groups can, indeed, help reduce the experience of social isolation, thereby bringing a sense of empathy and comfort to individuals, especially for vulnerable individuals in COVID-19 times ( Table 1 ). For instance, all over the world, children and adolescents are currently confined to their homes. Massive efforts are being made by teachers at schools and universities to create online courses and deliver them through TV broadcasts and the internet. In the event of home confinement, interaction could be enhanced via digital platforms, children could be involved in family activities, and self-sufficiency skills could be improved.^4 , 5^

**Table 1:** Virtual interventions to reduce social isolation in specific samples

Sample	Intervention proposal
General population	Online education, cultural courses, connections with friends, colleagues and family, psychological support
Children and adolescents	Online education, cultural courses, connections with friends, colleagues and family, shared games, psychological support
Elderly	Online education, cultural courses, connections with friends, colleagues and family, hobbies and enjoyable activities, psychological support
Health care workers	Online education, cultural courses, connections with friends, colleagues and family, psychological support
COVID-19 patients	Online education, cultural courses, connections with friends, colleagues and family, psychological support

Since the elderly represent the main risk group for the clinical complications of COVID-19, many countries have mandated older adults to self-isolate for a very long time. This initiative is expected to disproportionately affect elderly individuals whose only social contact occurs in places other than homes, such as at daycare venues, as well as community and religious centers. Although there may be disparities in the access to literacy on digital resources, the use of simple online technologies such as smartphones should be encouraged to promote more frequent contact with family and close friends, renew hobbies and participate in enjoyable activities without time constraints.^6 , 7^

Health care workers are also strongly affected by the COVID-19 pandemic.^8^ Due to overwhelmed hospitals, shortage of personal protective equipment and non-efficient governmental strategies, anger and frustration are common reactions.^9^ Thus, support from family and friends by means of communication technologies are essential to maintain good mental health. Likewise, patients are negatively affected by social isolation, suffering from sadness, uncertainty, and physical discomfort when hospitalized due to COVID-19. Additionally, poor health leads to communication difficulties as well as restricted use of smartphones by the patients. At-risk groups should be given adequate social and mental health support, which are needed and sometimes overlooked.^8^

Virtual communication paves the way for bringing people together and increases educational opportunities. Additionally, online cultural resources (e.g., films, arts, libraries, museums), educational courses, and psychosocial support programs are freely available for all individuals who have access to the internet. All these efforts aim to maintain a sense of continuity in personal and professional relationships in these difficult times.

In our view, helping communities, patients, vulnerable groups, and ultimately each of us, represents a backbone in the management of the current COVID-19 crisis. We acknowledge that quarantine is a necessary preventive measure during infectious disease outbreaks. In this regard, allowing quarantined people to connect with their friends and relatives using the internet, breaking social isolation, decreases loneliness and ultimately brings people together.
